# Effect of Probiotic E. coli Nissle 1917 Supplementation on the Growth Performance, Immune Responses, Intestinal Morphology, and Gut Microbes of Campylobacter jejuni Infected Chickens

**DOI:** 10.1128/iai.00337-22

**Published:** 2022-09-22

**Authors:** Yosra A. Helmy, Gary Closs, Kwonil Jung, Dipak Kathayat, Anastasia Vlasova, Gireesh Rajashekara

**Affiliations:** a Center for Food Animal Health, Department of Animal Sciences, Ohio Agricultural Research and Development Center, The Ohio State University, Wooster, Ohio, USA; University of California San Diego School of Medicine

**Keywords:** *C. jejuni*, probiotics, EcN, chicken, immune response, gut microbes, inflammatory response, intestinal morphology, antibodies

## Abstract

Campylobacter jejuni is the most common cause of bacterial foodborne gastroenteritis and holds significant public health importance. The continuing increase of antibiotic-resistant Campylobacter necessitates the development of antibiotic-alternative approaches to control infections in poultry and in humans. Here, we assessed the ability of E. coli Nissle 1917 (EcN; free and chitosan-alginate microencapsulated) to reduce C. jejuni colonization in chickens and measured the effect of EcN on the immune responses, intestinal morphology, and gut microbes of chickens. Our results showed that the supplementation of 3-week-old chickens daily with free EcN in drinking water resulted in a 2.0 log reduction of C. jejuni colonization in the cecum, whereas supplementing EcN orally three times a week, either free or microencapsulated, resulted in 2.0 and 2.5 log reductions of C. jejuni colonization, respectively. Gavaged free and microencapsulated EcN did not have an impact on the evenness or the richness of the cecal microbiota, but it did increase the villous height (VH), crypt depth (CD), and VH:CD ratio in the jejunum and ileum of chickens. Further, the supplementation of EcN (all types) increased C. jejuni*-*specific and total IgA and IgY antibodies in chicken’s serum. Microencapsulated EcN induced the expression of several cytokines and chemokines (1.6 to 4.3-fold), which activate the Th1, Th2, and Th17 pathways. Overall, microencapsulated EcN displayed promising effects as a potential nonantibiotic strategy to control C. jejuni colonization in chickens. Future studies on testing microencapsulated EcN in the feed and water of chickens raised on built-up floor litter would facilitate the development of EcN for industrial applications to control Campylobacter infections in poultry.

## INTRODUCTION

Campylobacter jejuni is the main cause of global bacterial foodborne gastroenteritis (1). It is one of the most prevalent causes of foodborne diseases in humans (over 800,000 cases annually), and also one of the leading causes of hospitalizations (more than 8,000 per year) in the United States (2). Campylobacter infections in humans are self-limiting and are characterized by watery and bloody diarrhea, fever, abdominal cramps, and nausea, and severe neurological consequences may also develop (3). Campylobacter infections are common in poultry, such as chickens, turkeys, ducks, and geese (4), and contaminated poultry products are the main sources of human infections. Infections in humans are sporadic and are associated with the improper handling of raw chicken or eating undercooked chicken products (1, 5).

Once Campylobacter is introduced into the flock, most of the birds within the flock get infected rapidly (6, 7). However, Campylobacter infections result in little to no clinical symptoms in poultry (6); but, colonization of Campylobacter in the intestinal tract leads to carcass contamination during the slaughter. The prevalence of Campylobacter can reach up to 100% in broiler flocks (6, 8) and can contaminate up to 100% of broiler carcasses (9–11). The increased prevalence of Campylobacter on-farm is associated with increased carcass contamination at processing (6, 7). Therefore, preharvest control of Campylobacter in chickens will result in a significant reduction in human infections (9, 12, 13).

Currently, Campylobacter infections in humans are treated with macrolides and fluoroquinolones when necessary. However, Campylobacter’s resistance to these groups of antibiotics has been reported (14, 15), which poses a threat to the effectiveness of existing antibiotic therapies in both medical and veterinary practices (16). Previous reports showed that 80%, 46%, 8%, and 100% of the Campylobacter isolated from chickens were resistant to tetracycline, erythromycin, ciprofloxacin, and penicillin, respectively (8, 15, 17). Additionally, there are no vaccines available to prevent Campylobacter colonization in poultry or to protect humans. Therefore, there is a critical need for antibiotic-alternative approaches (18) that can reduce Campylobacter prevalence, prevent the spread of antibiotic-resistant strains, and promote efficient poultry production.

E. coli Nissle 1917 (EcN) is a well-established probiotic bacterium that, when administered in an adequate quantity, confers host beneficial effects by facilitating mucosal repair and maintaining gut homeostasis (19). EcN lacks several virulence factors that are found in pathogenic E. coli strains and produces antimicrobial peptides which enhance the beneficial properties of EcN (20). EcN has been shown to work through (i) modulating host immune responses (21, 22), (ii) restoring gut barrier function, (iii) competitively excluding pathogens (19, 23, 24), and (iv) decreasing gut permeability and improving mucosal integrity (25–27). It has been reported that EcN reduces the colonization of Salmonella in the gut (28) and mitigates the invasion of epithelial cells by other pathogenic bacteria, such as Yersinia enterocolitica, Shigella flexneri, Legionella pneumophila, rotavirus, and Listeria monocytogenes, and also has been shown to possess antibacterial properties against enterohemorrhagic E. coli (EHEC) and C. jejuni *in vitro* (29–31). The efficacy and biosafety of EcN have been investigated in humans and in animal models to prevent ulcerative colitis, allergic dermatitis, inflammatory bowel diseases (IBD), and infant and neonatal calf diarrhea (19, 20, 23, 32). Our previous studies have shown that EcN possesses anti-Campylobacter properties *in vitro* and that EcN pretreatment of the human intestinal epithelial cells (HT-29) can protect the cells against C. jejuni invasion and intracellular survival through modulation of cellular integrity and the innate immune response (30, 33, 34). Here, we evaluated the ability of EcN (free and chitosan-alginate microencapsulated) to reduce C. jejuni colonization in chickens and measured the effect of EcN on the immune responses, intestinal morphology, and gut microbes of chickens.

## RESULTS

### Alginate-chitosan microencapsulation has no significant impact on the viability of EcN.

To evaluate the effect of the microencapsulation process on the viability of EcN and on the microencapsulation efficiency, the encapsulation yield of EcN was calculated. The average number of free EcN before microencapsulation was 1 × 10^9^ CFU/mL, while the average number of microencapsulated EcN was 9.6 × 10^8^ CFU/mL. The microencapsulation yield was calculated based on the formula

$$\text{Encapsulation yield }\left(\text{EY}\right)=\;\left(\text{N}/{\text{N}}_{0}\right)\;\times \text{ 1}00,$$

where N is the number of live bacteria (in CFU/g) contained in the microcapsules, and N_0_ is the number of viable bacteria (in CFU/mL) added during the production of the microcapsules.

**FIG 1:**
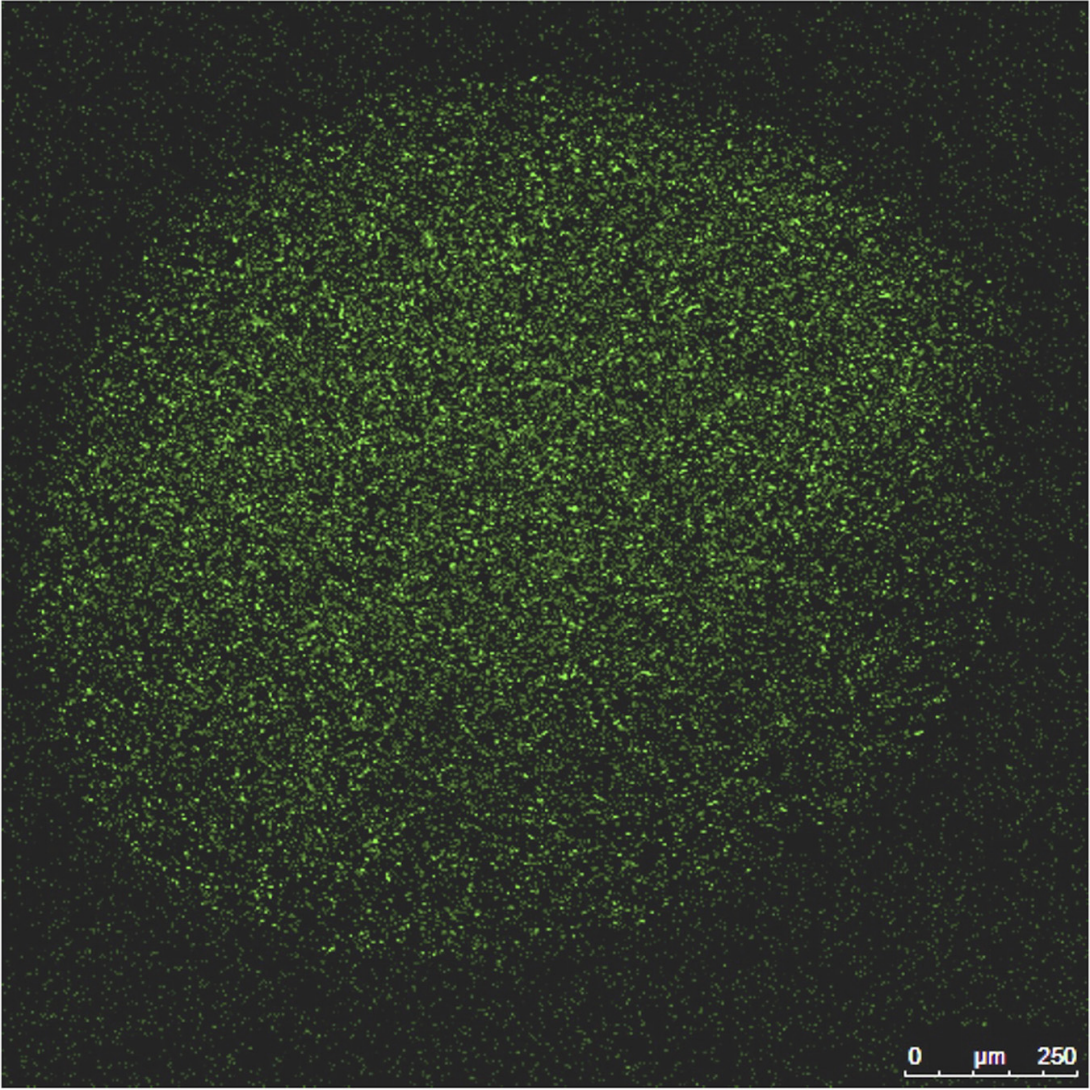
Confocal image of the chitosan-alginate microencapsulated EcN cells. The bacterial cells were prestained with 2 μM SYTO-9 fluorescent dye. The EcN cells were aggregated inside the chitosan-alginate microcapsule with clear margin. The size of the EcN microcapsules ranged between 700 and 1,000 μm.

The encapsulation yield of EcN in chitosan-alginate microcapsules was 96%. The EcN cells were aggregated inside the microcapsules, and the margins of the microcapsules were clearly demarcated. The average size of the EcN microcapsules ranged between 700 and 1,000 μm (Fig. 1).

### EcN (free/microencapsulated) reduced Campylobacter colonization in the chicken’s cecum.

**FIG 2:**
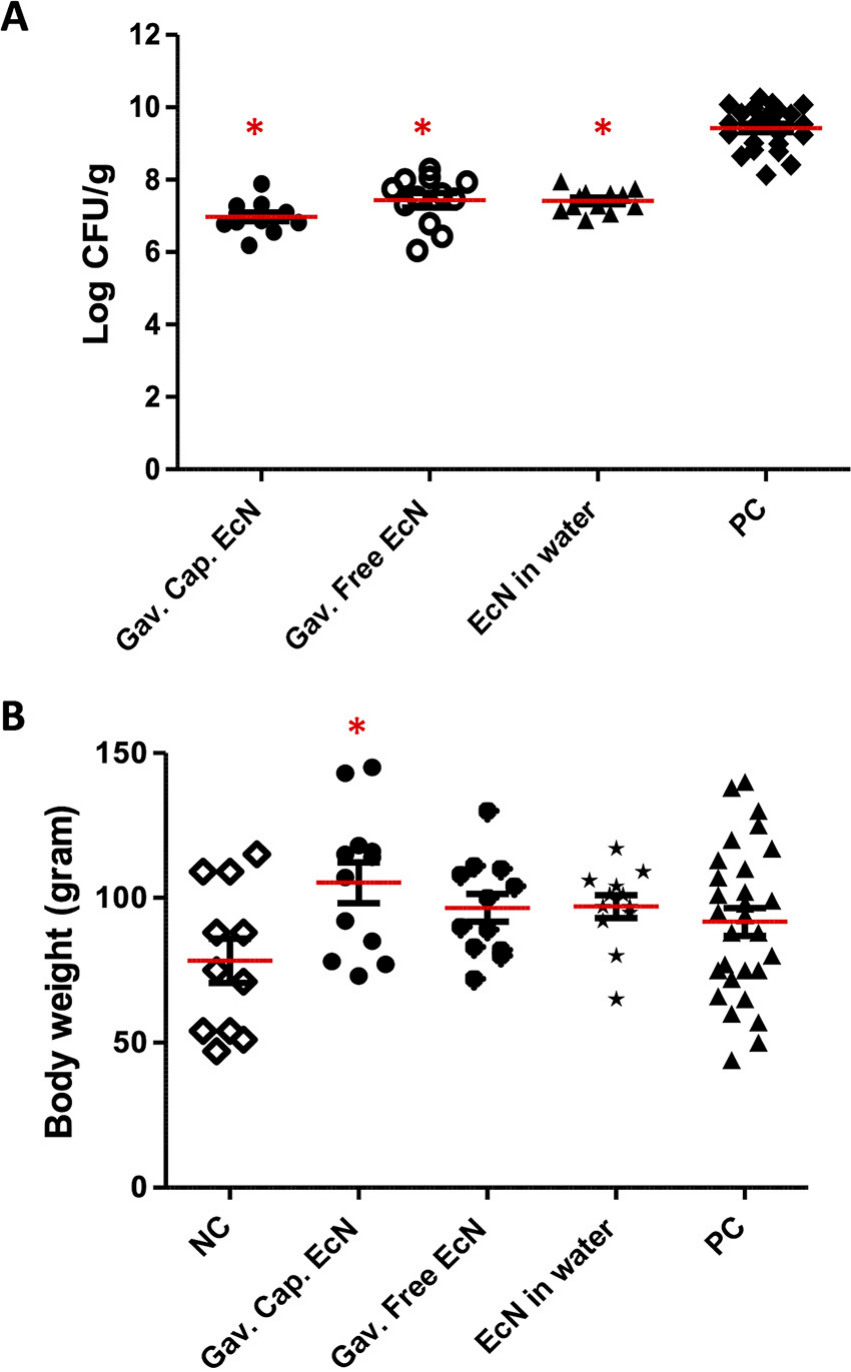
Effect of EcN treatment on C. jejuni colonization in chickens. Chickens were challenged at 4 weeks of age with a cocktail of 6 C. jejuni strains, including 5 chicken-associated field isolates (1 × 10^5^ per bird) and the C. jejuni 81-176 strain. The chickens were treated with EcN either daily in drinking water (free EcN) or using oral gavage (free or microencapsulated EcN) for 2 weeks from 3 to 5 weeks of age. Chicken cecum was collected at 5 weeks of age. Each dot represents the bacterial count for an individual chicken. *, *P* < 0.05 (statistically significant reduction of the C. jejuni population in ceca compared to the nontreated group (PC) by a one-way analysis of variance (ANOVA) with Tukey’s test).

Treatment of chickens with free EcN daily in drinking water for 2 weeks, starting 1-week prior to C. jejuni infection and ending 1-week postinfection (at 4 and 5 weeks of age), resulted in a 2.0 log CFU/g reduction of C. jejuni in the cecum compared to the non-treated, infected positive-control (PC) group (Fig. 2A) (*P* < 0.05). Similarly, the treatment of infected chickens, three times per week for 2 weeks (at 4 and 5 weeks of age) with free and microencapsulated EcN orally, resulted in 2.0 and 2.5 log CFU/g reductions, respectively, of C. jejuni in the cecum compared to the PC group (*P* < 0.05) (Fig. 2A). Interestingly, all of the treated chicken groups (free EcN in drinking water, oral free EcN, or microencapsulated EcN) showed higher body weights compared to the non-treated, non-infected negative-control (NC) or infected PC groups. Among the groups, orally administered microencapsulated EcN significantly increased the chickens’ body weights (*P* < 0.05) compared to the NC group (Fig. 2B).

To quantify EcN in the chicken’s cecum, EcN treatment was stopped 3 days before necropsy, and EcN-specific qPCR was performed on the total DNA extracted from the cecum. Our results showed that daily treatment of chickens with free EcN in drinking water for 2 weeks yielded up to 5 log CFU/g of EcN in the cecum. Further, the treatment of chickens orally with either free or microencapsulated EcN, three times weekly for 2 weeks, resulted in up to 4 log CFU/g of EcN and 2 log CFU/g (Fig. S1A) of EcN in the cecum, respectively. The standard curve used to quantify the EcN in the cecum is shown in Fig. S1B.

### Treatment of chickens with microencapsulated EcN did not impact the evenness and richness of the cecal microbiota.

The alpha diversity analysis revealed that oral gavage administration of chickens with free (*P* = 0.3; H = 0.9) and microencapsulated (*P* = 0.1; H = 2.08) EcN resulted in no statistically significant differences in the evenness and richness of the cecal microbiota compared to the PC group (*P* < 0.05) (Fig. S2). However, the treatment of chickens with free EcN in drinking water daily significantly increased the evenness and richness of cecal microbiota compared to the PC (*P* = 0.05; H = 3.9) and the NC (*P* = 0.01; H = 5.7) groups. The beta diversity analysis showed that the microbial community in the cecum of chickens treated with gavaged microencapsulated EcN was similar to that of the PC group (*P* = 0.4), whereas there was a dissimilar microbial community observed in the cecum of chickens gavaged with free EcN (*P* = 0.01) and with free EcN in drinking water (*P* = 0.02), compared to the PC group. Furthermore, spatial separation was observed between the free EcN treated group in water and the PC group (*P* = 0.001) as well as between the gavaged free EcN treated group and the NC and PC groups (*P* = 0.01), as determined by a principal coordinates analysis (PCoA) using the unweighted uniFrac data (Fig. S3).

**FIG 3:**
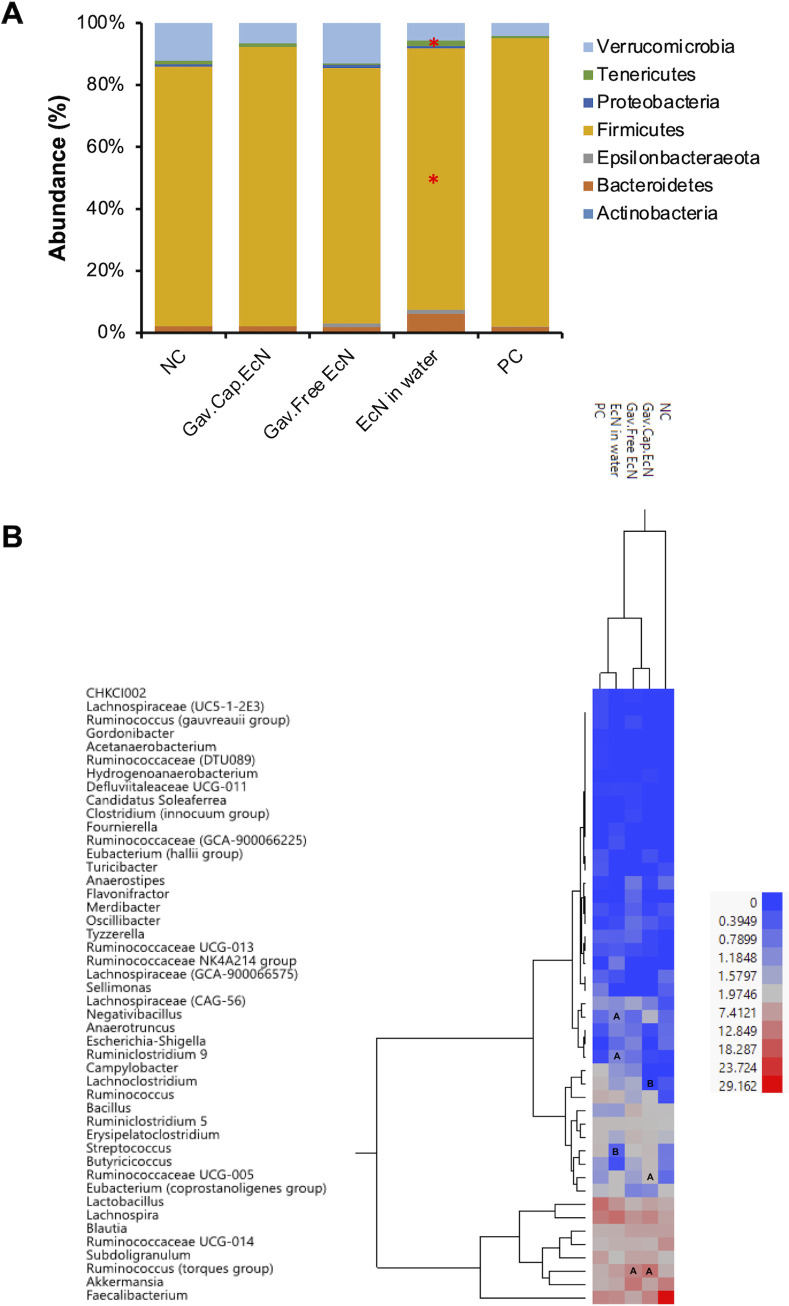
Impact of EcN treatment on the diversity and relative abundance of cecal microbiota at the (A) phylum level and the (B) genus level. Panels A and B in the heat map show whether the OTUs were significantly increased or decreased, respectively, compared to the infected and nontreated group (PC) (*P* < 0.05).

The predominant phylum present in the chicken cecum of all treated and control groups was Firmicutes (83.7% to 93.1%), followed by Verrucomicrobia (4.2% to 13%) and Tenericutes (0.5% to 1.7%). Compared to the PC group, the treatment of infected chickens with EcN in drinking water daily increased Bacteroidetes (1.5% to 6.1%; *P* < 0.05), Tenericutes (0.7% to 1.7%; *P* < 0.05), and Verrucomicrobia (4.2% to 5.7%), while reducing Firmicutes (93.1% to 84.5; *P* < 0.05). On the contrary, the treatment of chickens with free and microencapsulated EcN using gavage slightly increased Bacteroidetes [(1.5% to 1.8%) and (1.5% to 2.0%)] and Verrucomicrobia [(4.2% to 13.0%) and (4.2% to 6.5%)] while reducing Firmicutes [(93.1% to 82.5%) and (93.1% to 90.1%)], respectively, compared to the PC group (Fig. 3A). Interestingly, the treatment of infected chickens with microencapsulated EcN and EcN in water increased the abundance of Firmicutes in the cecum (83.7% to 90.1%) and (83.7% to 84.5%) compared to NC, respectively. However, at the phylum level, there was no significant change in the microbial community abundance in the cecum after the treatment with gavaged free or microencapsulated EcN compared to the PC group or between treated groups with all forms of EcN compared to the NC group (*P* < 0.05).

Additionally, at the genus level, the treatment of infected chickens with EcN in drinking water daily increased the abundance of *Ruminococcus* (torques group) (4.7% to 7.4%), *Negativibacillus* (0.7% to 1.1%; *P* < 0.05), *Ruminiclostridium* 9 (0.1% to 1.1%; *P* < 0.05), and *Akkermansia* (4.2% to 5.7%); while reduced the abundance of *Lactobacillus* (14.7% to 8.8%), Streptococcus (3.3% to 0.5%; *P* < 0.05), *Lachnoclostridium* (3.4% to 1.4%), *Subdoligranulum* (7.4% to 2.6%), and *Erysipelatoclostridium* (3.1% to 1.6%). Notably, the treatment of chickens with microencapsulated EcN using gavage increased the abundance of *Bacillus* (1.4% to 2.5%), *Blautia* (4.2% to 6.7%), *Ruminococcus* (torques group) (4.7% to 13.9%; *P* < 0.05), *Butyricicoccus* (1.4% to 3.1%), *Faecalibacterium* (10.8% to 11.3%), *Ruminococcaceae* UCG-005 (1.3% to 3.1%; *P* < 0.05), and *Akkermansia* (4.2% to 6.5%) in the cecum, whereas gavaged free EcN increased *Bacillus* (1.4% to 4.5%), *Blautia* (4.2% to 7.0%), *Ruminococcus* (torques group) (4.7% to 10.9%; *P* < 0.05), *Butyricicoccus* (1.4% to 1.6%), and *Akkermansia* (4.2% to 13.0%), compared to the PC group (Fig. 3B). Both gavaged microencapsulated and free EcN reduced the abundance of *Lachnoclostridium* ([3.4% to 0%; *P* < 0.05] and [3.4% to 1.7%], respectively), whereas gavaged free EcN reduced *Faecalibacterium* (10.8% to 5.9%). Interestingly, all of the EcN treated groups reduced *Lactobacillus* abundance in the cecum compared to the PC group; however, this reduction was not significant.

### EcN increased the villus height, crypts depth, and VH:CD ratio in the jejunum and ileum of treated chickens.

**FIG 4:**
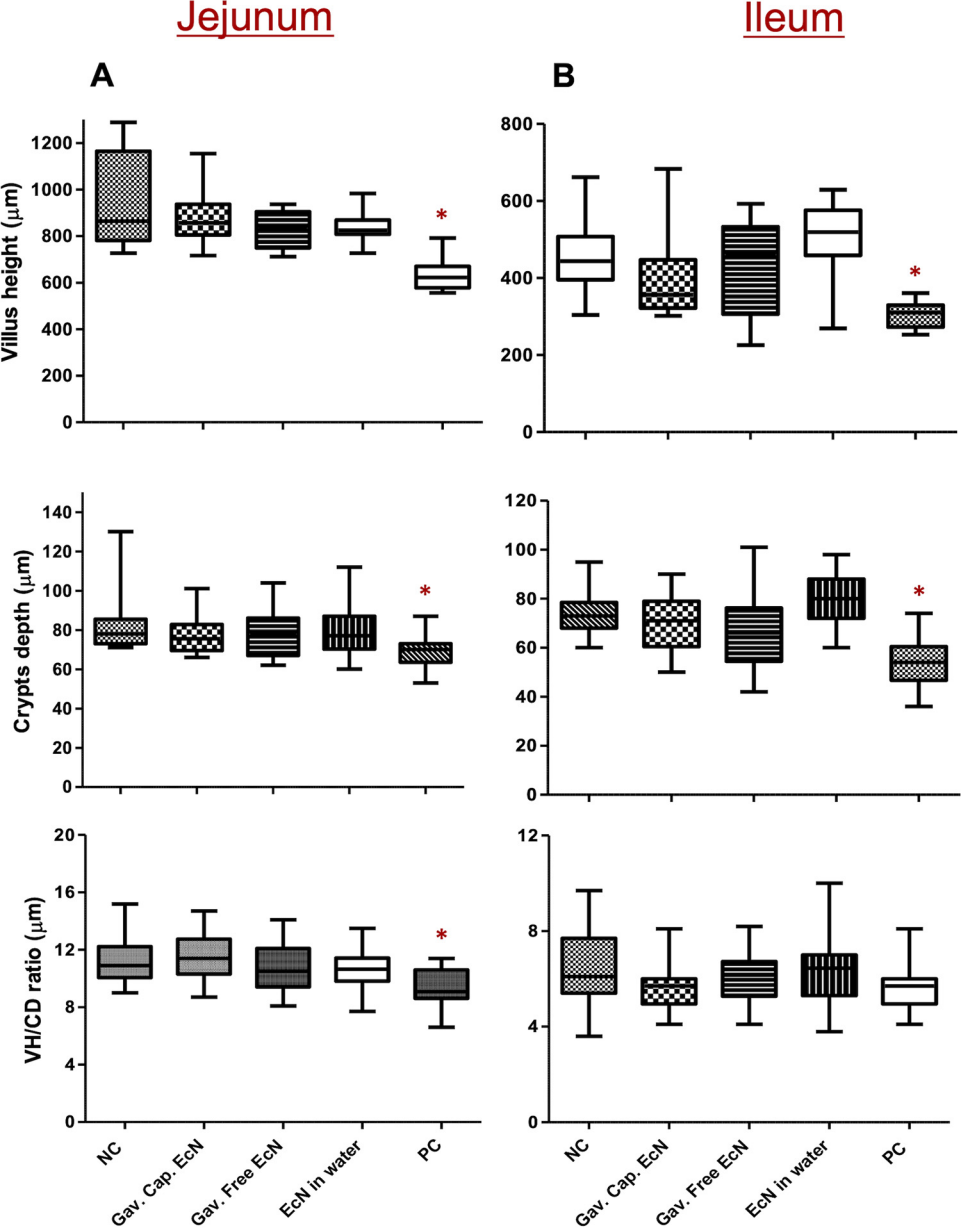
Effect of EcN treatment on the intestinal villus height, crypts depth, and the villus height to crypts depth ratio (VH:CD) of the (A) jejunum and (B) ileum of treated chickens. Samples from 12 chickens were included in each group. Approximately 2 cm of the ileum and jejunum were collected individually from each chicken and fixed in 10% neutral buffered formalin. The tissues were embedded in paraffin, and 3.5 μm sections were sliced and stained with hematoxylin and eosin (H&E). The villus height and crypt depth were determined using the NIH ImageJ program. *, *P* < 0.05 (significantly decreased villus height, crypt depth, and VH:CD ratio in the PC group compared to the noninfected, nontreated group [NC] by a one-way ANOVA with Tukey’s test).

To investigate the effect of EcN treatment and C. jejuni infection on the intestinal morphology, representative sections of the jejunum and ileum were collected from each chicken of the treated and control groups. Our results showed that the infection of chickens with C. jejuni significantly reduced the villus height in the jejunum (633.1 μm) and ileum (305.8 μm) compared to those of the NC group (944.9 μm and 453 μm, respectively; *P* < 0.05). C. jejuni infection also decreased (*P* < 0.05) the crypt depth in the jejunum (68 μm) and ileum (54 μm) compared to those of the NC group (82.3 μm and 70.2 μm, respectively) (Fig. 4A and 4B). Concurrently, there was a significant reduction (*P* < 0.05) in the VH:CD ratio of the jejunum (9.5) and ileum (5.7) in the PC group compared to the NC group (11.3 and 6.4, respectively) (Fig. 4A and 4B). Notably, treatment of chickens with different forms of EcN modulated the effect of C. jejuni infection on the villi height, crypt depth, or VH:CD ratio in the jejunum and ileum of the treated chickens compared to the PC group (*P* < 0.05) (Fig. 4A and 4B).

### EcN treatment increased the C. jejuni specific and the total IgA and IgY levels in the chicken serum.

**FIG 5:**
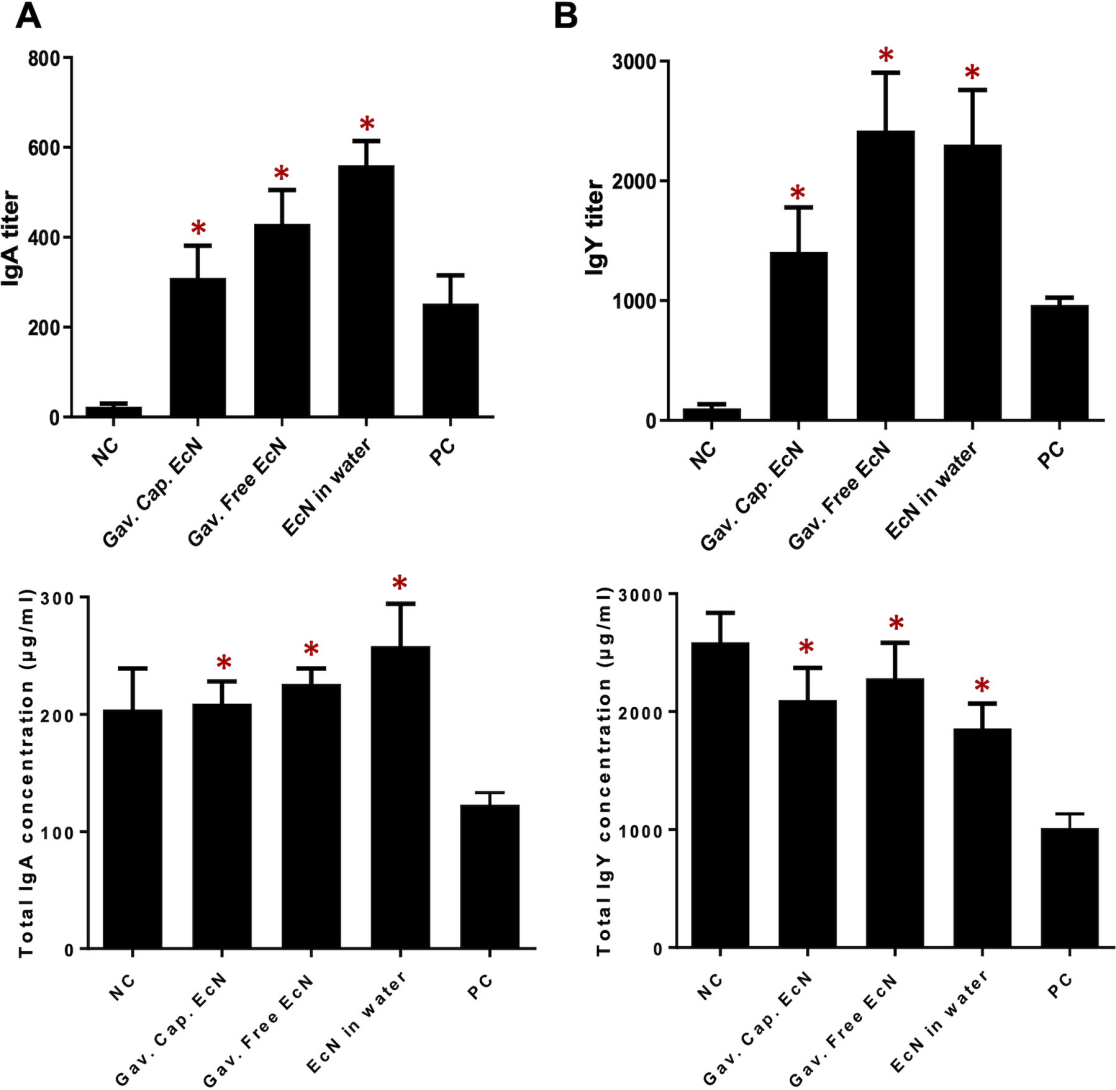
Concentration of the C. jejuni specific and total antibodies. (A) IgA and (B) IgY in the serum of infected chickens that were treated with EcN (free or microencapsulated). Samples from 12 chickens were included in each group. The concentrations of the antibodies were determined by enzyme-linked immunosorbent assay (ELISA) at 5 weeks of age. Free EcN treatment (gavaged or in drinking water) and microencapsulated EcN significantly increased the concentrations of total IgA and IgY in the serum, compared to the PC group. *, *P* < 0.05 (significantly increased serum antibodies in treated chickens compared to the control groups by a one-way ANOVA with Tukey’s test).

To study the effect of EcN treatments on the concentration of C. jejuni-specific IgA and IgY, an enzyme-linked immunosorbent assay (ELISA) was performed on serum at 5 weeks of age. Our results showed that the anti-C. jejuni IgA and IgY responses in the serum of chickens treated with free EcN either in drinking water or using oral gavage was higher than those treated with microencapsulated EcN. The treatment of chickens with microencapsulated EcN, gavaged free EcN, and free EcN in drinking water increased the C. jejuni specific IgA titer in the serum (310, 430, and 560, respectively), compared to that of the PC group (252.3) (*P* < 0.05) (Fig. 5A). Similarly, the treatment of chickens with microencapsulated EcN, gavaged free EcN, and free EcN in drinking water increased the C. jejuni specific IgY titer in the serum (1,408, 2,240, and 2,240, respectively), compared to that of the PC group (965) (*P* < 0.05) (Fig. 5B).

Additionally, our results revealed that the concentrations of the total chicken IgA and IgY were significantly higher in all EcN treated groups compared to those of the PC group (*P* < 0.05). Ths treatment of chickens with microencapsulated EcN, gavaged free EcN, and free EcN in drinking water increased the IgA concentration in the serum (209 μg/mL, 226 μg/mL, and 258 μg/mL, respectively), compared to the PC group (123 μg/mL) (*P* < 0.05) (Fig. 5A). Further, the concentration of IgY in all the three treatment groups was increased (2,099 μg/mL, 2,284 μg/mL, and 1,857 μg/mL, respectively), compared to the PC group (1,015 μg/mL) (*P* < 0.05) (Fig. 5B).

### Microencapsulated EcN induced the expression of cytokines and chemokines genes in the cecal tonsils.

The effects of C. jejuni (inflammatory) infection and EcN treatment (anti-inflammatory, protective) were evaluated by the quantification of the relative expression of cytokines and chemokines genes in the cecal tonsils. Our results showed that the treatment of the infected chickens with microencapsulated EcN using oral gavage (3 times per week for 2 weeks) significantly induced (*P* < 0.05) the expression of the Th17 pathway markers, which include cytokine genes such as IL-17A (3.5 fold), IL-17F (3.4 fold), and chemokine genes, such as Ch-CXCLI1 (2.0 fold) and CXCLI2 (ChIL-8; 3.3-fold), compared to that observed in the PC group (Table 1). Further, the Th1 pathway associated markers IFN-γ (2.4 fold) and IL-1β (3.2 fold) as well as the Th2 pathway associated markers IL-4 (2.4 fold) and IL-6 (4.3 fold) were also upregulated (*P* < 0.05). It is known that IL-6 and IL-1β promote the differentiation of Th17 cells, which thereby upregulates Th17 cytokines production (35). The microencapsulated EcN also increased the expression of the IL-10 gene (1.6 fold), which is generated by regulatory T-cells (Treg) to regulate the inflammatory effects of the Th cell responses. Moreover, the treatment of chickens with free EcN, either using oral gavage or in drinking water, upregulated the expression of different cytokine genes, including IL-4 (2.0 fold), IL-10 (1.2-1.8 fold), IL-17A (1.5-fold), IL-17F (1.5-1.6 fold), and IFN-γ (1.6-1.7 fold) (Table 1) (*P* < 0.05), while the upregulation of chemokine gene expression was not significant compared to that observed in the PC group. Interestingly, the infection of chickens with C. jejuni significantly upregulated (*P* < 0.05) the expression of the IL-10 (2.7 fold), IL-17A (2.9 fold), IL-1β (1.9 fold), IFN-γ (1.5 fold), and CXCLI1 (1.8 fold) genes compared to NC group (Table 1). The increase of IL-10, IL-4, IFN-γ, and IL-17A is an indication of activation of Th1, Th2, and Th17 responses; however, these are also associated with an increase in IL-10 gene expression. The Th1, Th2, and Th17-associated cytokines are involved in cellular and humoral immune responses and inflammatory responses (35).

**TABLE 1:** Effects of EcN treatment and C. jejuni infection on the expression of cytokine and chemokine associated genes^*a*^

Cytokine/Chemokine genes	Gavaged microencapsulated EcN^*b*^	Gavaged free EcN^*b*^	EcN in water^*b*^	PC^*c*^
IL-4	2.4	2.0	2.0	1.2
IL-6	4.3	1.0	1.0	1.0
IL-10	1.6	1.2	1.8	2.7
IL-17F	3.4	1.5	1.6	1.1
IL-17A	3.5	1.5	1.5	2.9
IFN-γ	2.4	1.7	1.6	1.5
IL-1β	3.2	1.1	1.2	1.9
Ch-CXCLI1	2.0	1.1	1.2	1.8
Ch-CXCLI2	3.3	1.2	1.1	1.2

a Data are presented as fold changes. A *P* value of ≤0.05 and a fold change of either ≥1.5 or ≤1.5 were used to determine significant differences in the each gene’s expression.

b Compared to the positive-control (PC; infected, nontreated).

c compared to the negative-control (NC; non treated, noninfected).

## DISCUSSION

Probiotic EcN has been reported to have a beneficial effect on host cells via the modulation of host immune responses (21, 22), the restoration of gut barrier function, the competitive exclusion of pathogens (19, 20, 23, 24), the maintenance of gut permeability, the improvement of mucosal integrity (25–27), and the reduction of the colonization of pathogens in the gut (28). Our previous studies showed that the EcN pretreatment of the human intestinal HT-29 cells can protect the cells against C. jejuni invasion and intracellular survival and that this efficacy of EcN is likely achieved through its effect on the cellular tight junction and innate immune response (30, 33, 34). In light of the above, the supplementation of poultry with EcN may reduce the risk of Campylobacter infection in poultry and reduce the risk of carcass contamination, which will have a significant impact on public health. Here, we evaluated the efficacy of EcN (free and chitosan-alginate microencapsulated) on C. jejuni colonization, gut health, and the immune responses of chickens. Our results showed that the treatment of chickens with free EcN daily in drinking water or free and microencapsulated EcN by oral gavage reduced C. jejuni colonization (2 to 2.5 log) in the chicken’s cecum (Fig. 2A). This was accompanied by increased body weight gain (Fig. 2A). Similarly, the treatment of infected chickens with Lactobacillus gasseri SBT2055 and Bifidobacterium longum PCB133 *per os* for 15 days reduced C. jejuni colonization in the cecum by 2 log and 1 log, respectively (36, 37), whereas the supplementation of Bacillus amyloliquefaciens in the diet for 42 days reduced C. jejuni colonization in the cecum by 1.7 log (38). Further, treatment with microencapsulated B. longum PCB133 + oligosaccharides for 14 days reduced C. jejuni colonization by up to 0.5 log (39). Interestingly, it was also reported that the mixing of different probiotic strains resulted in a better efficacy on reducing the Campylobacter in chickens. For example, the administration of *L. paracasei J.R +* L. lactis *Y + *L. rhamnosus 15b + L. lactis FOa in drinking water for 42 days reduced C. jejuni by 5 log in the duodena, ceca, and feces (40), while the administration of Enterococcus faecium *+ B. animalis +* L. reuteri *+* Pediococcus acidilactici *+ L. salivarius* in drinking water for 14 days reduced C. jejuni colonization by up to 5.5 log in the cecum (41). Our study suggests that EcN can be used to reduce C. jejuni infection in preharvest poultry as well to promote growth performance of flocks.

Gut microbiota play critical roles in the maintenance of chicken intestinal health by modulating physiological functions that are required to maintain the intestinal homeostasis of the host (42). Probiotics were reported to improve chicken growth performance and feed efficiency through the maintenance of a beneficial microbial population, improvement of feed intake, and alteration of bacterial metabolism (43). They improve the gut microbial balance by preventing bacterial colonization, immune stimulation, and competitive exclusion, which contributes to keeping the host healthy (44) and maintaining a beneficial microflora that suppresses the growth of pathogens (45). In this study, the treatment of chickens with gavaged free and microencapsulated EcN did not impact the evenness and richness of the cecal microbiota compared to those of the PC group, and the microbial community in the cecum of chickens treated with microencapsulated EcN was similar to that of the PC group (Fig. S2; S3). A dysbiotic microbial community is associated with the modulation of the host immune system and the intestinal inflammatory responses, leading to the alteration of the gut mucosal epithelium and, consequently, colonization by pathogens (46). Interestingly, the treatment of chickens with microencapsulated EcN increased the abundance of Firmicutes (83.7% to 90.1%) compared to that observed in the NC group. A similar result was obtained when chickens were supplemented with Lacticaseibacillus rhamnosus GG or other different mixtures of probiotics, such as Pediococcus pentosaceus, B. cereus, B. macerans, B. subtilis, L. plantarum, and Issatchenkia orientalis (47, 48). The high Firmicutes abundance in the gut positively correlated with feed efficiency and the chickens’ performance (49, 50). Therefore, we suggest that the increase in the chicken’s body weight in the EcN treated groups (Fig. 2B) might be due to the high abundance of Firmicutes in the cecum. Additionally, gavaged microencapsulated and gavaged free EcN increased the abundance of *Bacillus* ([1.4% to 2.5%] and [1.4% to 4.5%]); and *Butyricicoccus* ([1.4% to 3.1%] and [1.4% to 1.6%]), respectively (Fig. 3B). *Butyricicoccus* plays a role in cell permeability and intestinal barrier functions (51). B. pullicaecorum has been shown to reduce Salmonella, Campylobacter, and Clostridium perfringens infections in chickens (52, 53). In a similar study, a *Bacillus*-based probiotic increased the abundance of *Butyricicoccus* in the guts of chickens infected with Salmonella (54). We suggest that the anti-C. jejuni activity of EcN might be due to its growth-promoting effect on *Butyricicoccus*.

The healthy intestinal mucosa is well-differentiated and contains long intestinal villi with a high VH:CD ratio (55). It was reported that infections of broiler chickens with Campylobacter decreased the villus height, villus surface area, and crypt depth at 21 days of age (56). Similarly, our study showed that the infection of chickens with C. jejuni significantly reduced the villus height, crypt depth, and VH:CD ratio in the jejunum and ileum of infected chickens at 5 weeks of age (Fig. 4A and 4B). Campylobacter infection reduced villus length, which might reduce nutrient absorption, increase secretion in the gastrointestinal tract, and, consequently, reduced body weight gain (57). Interestingly, the treatment of chickens with EcN moderated the effect of C. jejuni infection on the intestine by increasing the villus height, crypt depth, and VH:CD ratio in the ileum and jejunum, suggesting an increased surface area capable of more efficient absorption of nutrients, leading to more efficient feed utilization. These results are in line with earlier reports that investigated the effect of feeding chickens with B. subtilis, E. faecium, L. reuteri, B. thermophilum or a mixture of L. acidophilus *+* L. plantarum *+* E. faecalis (58–61). The increased villus height and the VH:CD ratio are also related to the increased turnover of crypt cells (43), and the increased villus height may also be a result of activated cell mitosis in the crypt (58).

The infection of the gut by pathogenic bacteria, including C. jejuni, is recognized by the host immune system, which consequently responds through complex connected pathways involving the innate and adaptive immune systems (57). The administration of probiotics, including EcN, also plays a vital role in regulating the production of cytokines and chemokines, which in turn regulates immunity against pathogens (62). Our results showed that the treatment of chickens with EcN induced cytokine and chemokine gene expression. Specifically, this induction was significant with the use of microencapsulated EcN (1.6 to 4.3-fold) compared to the results observed in the PC group (Table 1), which can consequently activate the Th1, Th2, and Th17 pathways. The pretreatment of human intestinal epithelial cells with EcN promotes immune activation and induces the production of anti-inflammatory mediators, which is consistent with the results obtained in this study in chickens (30, 33, 34). Similarly, *Lactobacilli* was reported to induce the Th-1 and Th-2 immune responses (63, 64). The stimulation of the immunoregulatory response via the activation of CD4 T-cell pathways is thought to be important in limiting the invasion and colonization of C. jejuni in the chicken gastrointestinal tract (65). Our data imply that the treatment of chickens with EcN regulates the expression of genes involved in the protective innate immune response and induces the expression of anti-inflammatory cytokine genes (IL-6, IL-10, IL-17A, and IL-17F) that are likely to protect the host against a C. jejuni-induced proinflammatory response (66). These findings reveal potential mechanisms of EcN for mediating the reduction of cecal colonization with C. jejuni.

One of the major findings of our study is that EcN administration appears to enhance the production of serum immunoglobulin antibodies (both C. jejuni-specific and the total IgA and IgY) in chickens (Fig. 5). These serum immunoglobulins serve as essential indicators of humoral immune status, owing to their critical roles in immune function and infection resistance (67, 68). It was reported that supplementation with B. subtilis increased IgA and IgY concentrations in the broilers’ serum at 42 days of age (69, 70), while the supplementation of a mixture of L. acidophilus, B. subtilis *DSM 17299*, and *C. butyricum* increased the concentrations of IgM and IgA in the serum of broilers and did not affect the concentration of IgY (71). Furthermore, the coadministration of an N-glycan-based C. jejuni vaccine with L. reuteri or Anaerosporobacter mobilis elevated the level of IgY in the broilers’ serum at 35 days of age (72). The improved serum immunoglobulin of chickens supplemented with probiotics in this study might be due to the immunomodulatory effect of EcN (73). Therefore, the ability of EcN to enhance the immune system efficiency is beneficial for its use as an antibiotic-alternative to improve animal health and productivity.

In conclusion, probiotic EcN (free or microencapsulated) reduced C. jejuni colonization in infected chickens when administered either using oral gavage or in drinking water. However, this reduction was higher when the chickens were treated with microencapsulated EcN. Furthermore, EcN improved the intestinal morphology, increased the C. jejuni-specific as well as the total IgA and IgY, and enhanced the immune responses of treated chickens via the activation of the Th1, Th2, and Th17 pathways, which likely contributed to protection against C. jejuni infection. The improved performance of broiler chickens administered with EcN may be associated with the improved intestinal morphology as well as the changes in the gut microbiome, such as the increase in the abundance of the phylum Firmicutes and the genus *Butyricicoccus*. Overall, microencapsulated EcN provided better anti-C. jejuni protection and displayed a promising effect as a potential nonantibiotic approach to control C. jejuni infections in chickens, thus enhancing food safety, which can eventually reduce the risk of campylobacteriosis in humans. In the future, we will focus on evaluating the impact of microencapsulated EcN, when administered in feed or in water, on Campylobacter infections in chickens raised under field-simulated conditions.

## MATERIALS AND METHODS

### Bacterial strains and culture conditions.

E. coli Nissle 1917 (EcN) was provided by Ulrich Sonnenborn (the Department of Biological Research, the Ardeypharm GmbH, Germany) and was cultured in Luria-Bertani (LB; BD Difco, NJ, USA) media at 37°C. The logarithmic-phase grown EcN was harvested via the centrifugation of the culture at 5,000 × *g* for 10 min. The EcN cells were then washed (2×) using phosphate-buffered solution (PBS) and realiquoted in 0.1% peptone. The optical density was adjusted to an OD_600_ = 1.0 (1 × 10^9^ CFU/mL). The EcN cell suspension was split into two parts. The first part was utilized for the preparation of microencapsulated EcN, and the second part served as a non-microencapsulated control (free EcN). The well-characterized and highly invasive C. jejuni 811-76 strain (ATCC BAA-2151) and 5 precharacterized C. jejuni chicken isolates (74, 75) were used for challenge studies in chickens. C. jejuni strains were cultured microaerobically (85% N_2_, 10% CO_2_, 5% O_2_) on CHROMagar Campylobacter (DRG International Inc., NJ, USA) at 42°C for 48 h (76).

### Microencapsulation of EcN.

The microencapsulated EcN was prepared as described previously (33). EcN was grown to the logarithmic phase, washed (2×) with PBS, and resuspended in 5 mL of 0.1% peptone. 10 milliliters of 2% sonicated sodium alginate (at 40 MHz amplitude for 15 min) solution (Fisher Scientific, MA, USA) was added to the mixture (1:2 vol/vol). The EcN-sodium alginate mixture was then placed in a 1 mL syringe (30-gauge needle) and dispensed drop by drop into 10 mL of 0.05 M CaCl_2_ containing Tween 20 (0.1%; a surfactant) to form encapsulated beads. The beads in CaCl_2_ solution were incubated at room temperature for 30 min and then washed (2×) with PBS. The beads were then coated with 0.1% of chitosan (1 g/1 mL of water; wt/vol) (MP Biomedicals, CA, USA) at a pH ranging between 5.7 and 6.0. Chitosan was prepared by sonication at 40 MHz amplitude for 20 min in a sonicator (Sonics & Materials Inc., Vibra-Cell) and autoclaved for 15 min at 121°C. The beads and chitosan mixture were incubated at room temperature with shaking at 100 rpm for 40 min. The coated microcapsules were washed with PBS and kept in 0.1% peptone at 4°C until needed.

The viable EcN counts in the microcapsules and the encapsulation yield were determined as described previously (33). One gram of microencapsulated EcN was resuspended in a solution of 1% sodium citrate dihydrate and stirred for 5 to 10 min. Then, 10-fold serial dilutions were plated on LB agar plates. The plates were incubated at 37°C for 24 h, and the bacteria were counted as CFU/g.

### Determination of EcN microcapsule size.

EcN was grown in LB broth overnight at 37°C, centrifuged for 10 min at 5,000 × *g*, and the pellet was resuspended in PBS. EcN was stained with 2 μM SYTO 9 (Thermo Fisher Scientific, MA, USA) for 10 min, centrifuged (at 5,000 × *g* for 10 min), and washed (5×) with PBS (33). The stained cells were resuspended in 0.1% peptone and the microcapsules were generated as above. The microencapsulated EcN was investigated using a confocal laser scanning microscope (Carl Zeiss, USA) with the laser power set at 81.2, an excitation wavelength of 490 nm, and an emission wavelength of 525 nm.

### Effect of free and microencapsulated EcN on C. jejuni infections in chickens.

Three-week-old specific-pathogen free (SPF) chickens were obtained from a flock free of Campylobacter and Salmonella at the Center for Food Animal Health, The Ohio State University. Before starting the experiment, cloacal swabs were randomly collected from three chickens in each group, and the samples were confirmed negative for Campylobacter by plating on CHROMagar (75, 77–79). Chickens were grown under isolated conditions and provided with feed and water *ad libitum* (77). The experiment was conducted in two separate trials, and the nontreated groups from both trials were combined. The chickens (*n* = 12/group) were divided into five groups: (i) treated with encapsulated EcN three times per week orally for 2 weeks, (ii) treated with free EcN three times per week orally for 2 weeks, (iii) treated daily with free EcN in drinking water for 2 weeks, (iv) nontreated and infected with C. jejuni (PC; positive control), and (v) noninfected, nontreated (NC; negative control). For groups 1 and 2, microencapsulated (9.6 × 10^8^ CFU/bird) and free EcN (1 × 10^9^ CFU/bird) were suspended in water, and each bird received 1 mL of the solution. The first 3 doses were inoculated 1 week before the challenge, and these were followed by another 3 doses after the challenge, with a 1-day interval between each treatment. For group 3, the chickens were administered daily with free EcN (1 × 10^9^ CFU/mL) in drinking water. All treatment groups (1, 2, and 3) were administered with EcN for 2 weeks (at weeks 4 and 5 of age). Chickens were challenged orally at 4 weeks of age with a cocktail of 6 C. jejuni strains (a mixture of C. jejuni 81-176 and five genetically diverse chicken associated field isolates [74, 75]) at a concentration of 1 × 10^5^ CFU/bird aliquoted in Mueller-Hinton (MH) broth. Following treatment (at 5 weeks of age), the chickens were necropsied, and one cecum was collected aseptically, homogenized in PBS, 10-fold serially diluted, and plated on CHROMagar. The plates were then incubated for 48 to 72 h at 42°C in microaerophilic conditions as described previously (75, 80). The other cecum was flash-frozen in liquid nitrogen and used for the microbiota studies.

### Genomic DNA extraction, 16S rRNA sequencing, and bioinformatic analysis.

To determine the effect of EcN treatment on the gut microbial community, genomic DNA was extracted from the chicken cecum of the treated and the control groups. Cecum was collected, flash-frozen immediately in liquid nitrogen, and frozen at −80°C until needed. About 0.25 g of the snap-frozen cecal content was used to extract the DNA using a DNA Purification Kit (PureLink Microbiome; Thermo Fisher Scientific, MA, USA). The traces of RNA were removed using 100 mg/mL of RNase (Thermo Fisher Scientific, MA, USA). The quantity and quality of the DNA were determined using a NanoDrop 2000 C Spectrophotometer (Thermo Fisher Scientific, MA, USA). For the 16S rRNA V4-V5 variable region sequencing, about 15 ng of pure DNA was used (77, 81). The amplicon libraries were prepared using a Ready Mix PCR Kit (IFU KAPA HiFi HotStart, Roche Sequencing and Life Science, MA, USA), and PCR cleanup was conducted using Agincourt AMPure XP beads (Beckman Coulter, CA, USA). The 16S sequencing and the bioinformatic analysis were performed as described previously (82, 83).

### EcN quantification in the gut.

The colonization of EcN in the chickens’ cecum was assessed via EcN-specific quantitative PCR (qPCR) on the genomic DNA extracted above (*n* = 6/group) using primers (EcN1: 5′-GCATTCGCCCCAGAGGAATAA-3′, EcN2: 5′-GTGTGCCTGAGACCCCAACAT-3′) as described previously (84). The qRT-PCR was conducted using qPCR Master Mix (SensiMix SYBR Hi-ROX), according to the manufacturer’s instructions (Thomas Scientific, NJ, USA), in a RealPlex^2^ Mastercycler (Eppendorf, CT, USA), using an annealing temperature of 55°C. The standard curve of EcN qRT-PCR was used to enumerate the EcN colonization. The standard curve was generated by the qPCR of 10-fold serially diluted DNA extracted from an EcN pure culture (OD_600_ of 1.0) using a MasterPure DNA Purification Kit (Thermo Fisher Scientific, MA, USA).

### Effect of EcN treatment on the intestinal morphology of chickens.

Approximately 2 cm of the ileum and jejunum were collected individually from each chicken of the treated and control groups and fixed in 10% neutral buffered formalin for gut morphological measurements (85, 86). The tissue was paraffin-embedded, and 3.5 μm sections were made and stained using hematoxylin and eosin (H&E). The stained tissue was assessed microscopically. The villus height and crypt depth were measured using the NIH ImageJ program. The villus height was detected by measuring the distance between the villus tip and the crypt opening, while the crypt depth was determined by measuring the distance between the crypt base and the level of the opening of the crypt. The villus height to crypt depth (VH:CD) mean ratios were calculated.

### C. jejuni-specific IgA and IgY concentrations in chicken serum.

The C. jejuni-specific IgA and IgY concentrations in chicken serum were measured using an enzyme-linked immunosorbent assay (ELISA). The outer membrane protein (OMP) was prepared by growing the 6 C. jejuni strains on CHROMagar plates at 42°C for 48 h under microaerobic conditions. The bacteria were collected in PBS and centrifuged at 6,000 × *g* for 15 min at 4°C. The pellet was aliquoted in PBS with 1 mM phenylmethylsulfonyl fluoride (Millipore Sigma, MO, USA) as a protease inhibitor and sonicated (using 3 pulse and 4 amplitude) 15 times for 20 s. The sonicated bacteria were centrifuged at 6,000 × *g* for 10 min at 4°C, and the supernatant was then centrifuged again at 10,000 × *g* for 2 h at 4°C. The pellets were collected, suspended in 100 μL of PBS, and stored at −20°C until needed. The concentration of the protein was measured using the Quick Start Bradford Protein Assay Kit (Bio-Rad, Hercules, CA) according to the recommendations from the manufacturer.

The ELISA was conducted as described previously (87). Briefly, the extracted OMPs from all C. jejuni strains were mixed at an equal concentration (1/1; vol/vol). The flat-bottom, 96-well microtitration plates (Thermo Fisher Scientific, MA, USA) were coated with an OMP mixture (at a concentration of 50 μg/mL; 50 μL/well), suspended in 0.05 M carbonate buffer (coating buffer) at a pH of 9.5, and incubated overnight at 4°C. The plates were then washed (3×) with PBS containing 0.1% Tween 20 (PBS-T; 200 μL). 200 μL of 5% nonfat dry (skim) milk (NFDM) in PBS-T (blocking buffer) was added to each well, and the plate was then incubated for 2 h at 37°C. The plates were then washed again (3×) with PBS-T. Serum samples (50 μL) were added to each well in duplicate with a 4-fold serial dilution, starting from 1:10 for IgA and 1:64 for IgY diluted in 5% NFDM blocking solution and then incubated at 37°C for 1 h. The plates were then washed (3×) with PBS-T. 50 μL of anti-IgA (0.34 μg/mL; 1:3000; ABCAM, MA, USA) or anti-IgY (0.5 μg/mL; 1:2000; ABCAM) diluted with 5% NFDM blocking solution were added to each well, and the plates were then incubated at 37°C for 30 min. The plates were then washed (3×) with PBS-T. After 10 min of color development, 50 μL of peroxidase substrate solution were added to each well, and the reaction was stopped by adding 50 μL of 1N HCl solution. The optical density (OD) was measured at 450 nm in a microplate ELISA reader.

### Total IgA and IgY concentrations in chicken serum.

For measuring the concentration of IgY, 96-well ELISA plates were coated with Goat Anti-Chicken IgY H&L (ABCAM, MA, USA) at 5 μg/mL concentration in 0.05 M carbonate buffer (coating buffer) at a pH of 9.5 and incubated overnight at 4°C. The plates were washed (3×) with PBS-Tween 20 (200 μL). 200 μL of 5% nonfat dry (skim) milk (NFDM) in PBS-T was added to each well, and the plate was then incubated at 37°C for 2 h. The plates were then washed again (3×) with PBS-T. Serum samples (100 μL) were added to each well in duplicate with a 1:1000,000 dilution in 5% NFDM blocking solution. The samples were then incubated at 37°C for 1 h. A standard of known concentration, normal chicken purified IgY antibody was included (Sigma-Aldrich). The plates were then washed (3×) with PBS-T. 50 μL of anti-IgY (1:18000; ABCAM) diluted with 5% NFDM blocking solution were added to each well, and the plates were then incubated at 37°C for 30 min. The plates were then washed (3×) with PBS-T. After 10 min of color development, 50 μL of peroxidase substrate solution was added to each well, and the reaction was then stopped by adding 50 μL of 1N HCl solution. The optical density (OD) was measured at 450 nm in a microplate ELISA reader. The concentration of the total IgA was measured using an IgA chicken ELISA kit (ABCAM, MA, USA), following the recommendations of the manufacturer.

### Quantitative real-time reverse transcription-PCR (qRT-PCR).

The effect of EcN treatment and C. jejuni infection on the cytokine- and chemokine-associated gene expression was determined using qRT-PCR. Cecal tonsils were collected individually from each chicken in RNAlater, stored at 4°C for 1 week. Then, the RNAlater was discarded, and the cecal tonsils were stored at −80°C until further use. Total RNA was extracted from each cecal tonsil using the miRNeasy Minikit (Qiagen, MD, USA), and the residual DNA was removed using a genomic DNA removal mixture (Qiagen, MD, USA). The quality and quantity of the RNA were determined using a NanoDrop 2000 C spectrophotometer (Thermo Fisher Scientific, MA, USA). 5 μg of the pure RNA was utilized to synthesize the cDNA using the Qiagen RT^2^ First Strand Kit (Qiagen, MD, USA) (88). The qRT-PCR was performed using qPCR Master Mix (SensiMix SYBR Hi-ROX; Thomas Scientific, MA, USA) in a RealPlex^2^ master cycler (Eppendorf, CT, USA) and an annealing temperature of 55°C. Gene-specific primers were synthesized from Integrated DNA Technologies (IDT) (57). The threshold cycle (Ct) value was calculated for each gene and standardized to glyceraldehyde-3-phosphate dehydrogenase (GAPDH), a housekeeping gene. The fold changes in the expression of the gene between the different EcN treated samples and the control samples were calculated using the ΔΔCt method (88). Two independent repeats were conducted.

### Statistical analyses.

The statistical analysis of the C. jejuni colonization data, intestinal morphology, and C. jejuni-specific IgA and IgY concentrations was done on GraphPad Prism 5.0 software (GraphPad) using a one-way analysis of variance (ANOVA) with Tukey’s test. A two-way ANOVA was used to analyze the qRT-PCR data. Statistically significant differences in the expression of the genes were determined using a *P* value of ≤0.05 and a fold change of ≥1.5 or ≤1.5. The Kruskal-Wallis and permutational ANOVA (PERMANOVA) tests were used to analyze the alpha and beta diversities of the gut microbes (*P* < 0.05), respectively. The OTU relative abundance differences between the EcN treated groups and the control groups were calculated using a Mann-Whitney *U* test. The statistically significant differences between the means were determined using a *P* value of <0.05.

### Ethical statement.

All of the experimental procedures were carried out in accordance with the Institutional Animal Care and Use Committee (IACUC) guidelines (accredited by the Association for Assessment and Accreditation of Laboratory Animal Care International) of The Ohio State University under IACUC protocol number 2010A00000149.

### Data availability.

The data generated or analyzed in this study are included in this published article and in the supplementary files. The microbiome sequence data have been deposited in the BioProject database under accession number PRJNA766997.
