# Supplementary material for: Effect of Probiotic E. coli Nissle 1917 Supplementation on the Growth Performance, Immune Responses, Intestinal Morphology, and Gut Microbes of Campylobacter jejuni Infected Chickens
Source: Infect Immun. 2022 Sep 22;90(10):e00337-22. doi: 10.1128/iai.00337-22 (PMC9584303; doi:10.1128/iai.00337-22)
Supplement: Supplemental file 1 — Fig. S1 to S3. Download iai.00337-22-s0001.pdf, PDF file, 0.3 MB [file iai.00337-22-s0001.pdf]

Supplementary figure legends

Fig. S1. A) Quantification of EcN in chicken's cecum. Samples from six chickens were included in each group, $*P<0.05$, and B) The standard curve used to measure the level of EcN in the cecum. Amount of EcN in chicken's cecum was assessed using EcN-specific qPCR. The standard curve of EcN qRT-PCR was used to quantify the EcN by log CFU.

Fig. S2. Shannon's diversity index showing the microbial richness and evenness in EcN treated groups compared to infected, non-treated group (PC) group. Gavaged free (P=0.3; H=0.9) and microencapsulated (P=0.1; H=2.08) EcN caused no significant differences in the richness and evenness of cecal microbiota compared to PC group. $*P<0.05$, Kruskal-Wallis test.

Fig. S3. Impact of free EcN in drinking water, gavaged free EcN and gavaged microencapsulated EcN treatments on beta diversity of cecal microbiota of chickens. Beta diversity was evaluated using Principal coordinate analysis of unweighted uniFrac values. Each dot represents one cecum sample from treated or control group. Blue circle: infected chicken group, treated with gavaged microencapsulated EcN. Red circle: infected chicken group, treated with free EcN in drinking water. Orange circle: infected chicken group, treated with gavaged free EcN. Purple circle: infected, non-treated chicken group (PC). Green circle: non-infected, non-treated chicken group (NC).

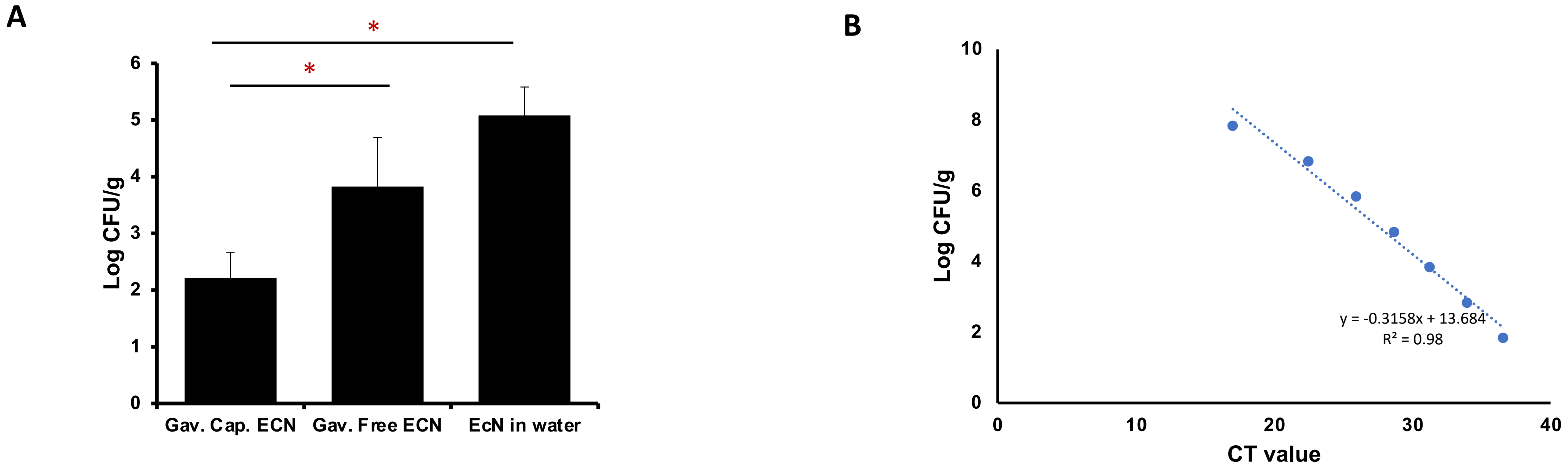
A
*
*
Log CFU/g
0
1
2
3
4
5
6
Gav. Cap. ECN
Gav. Free ECN
EcN in water
B
Log CFU/g
0
2
4
6
8
10
y = -0.3158x + 13.684
R² = 0.98
0
10
20
30
40
CT value

Figure S1

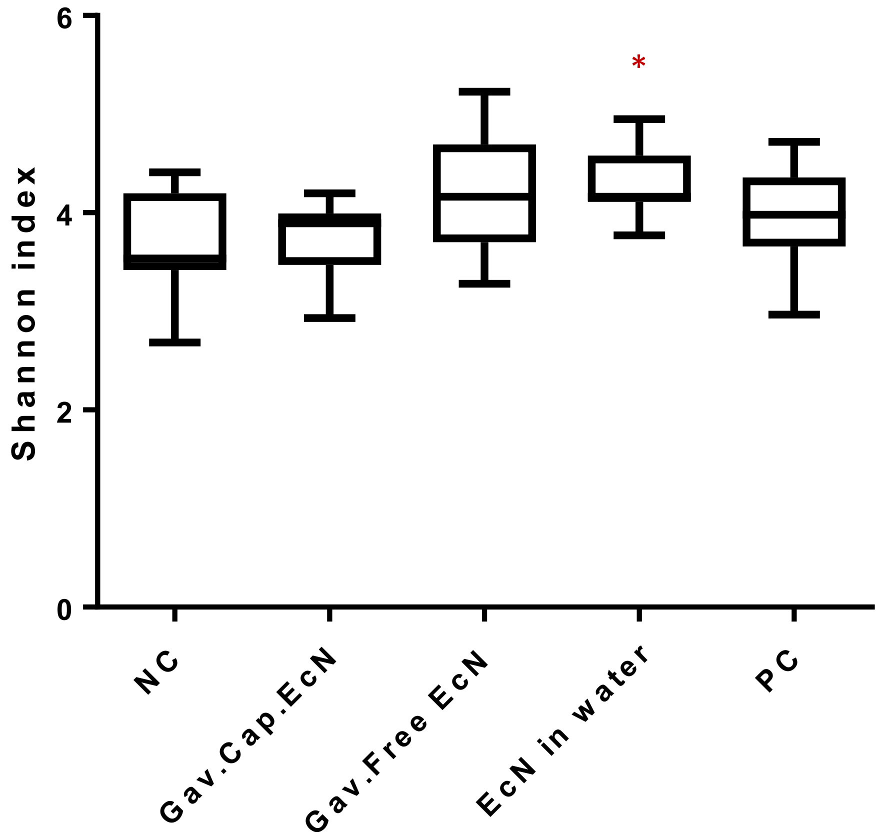

Figure S2

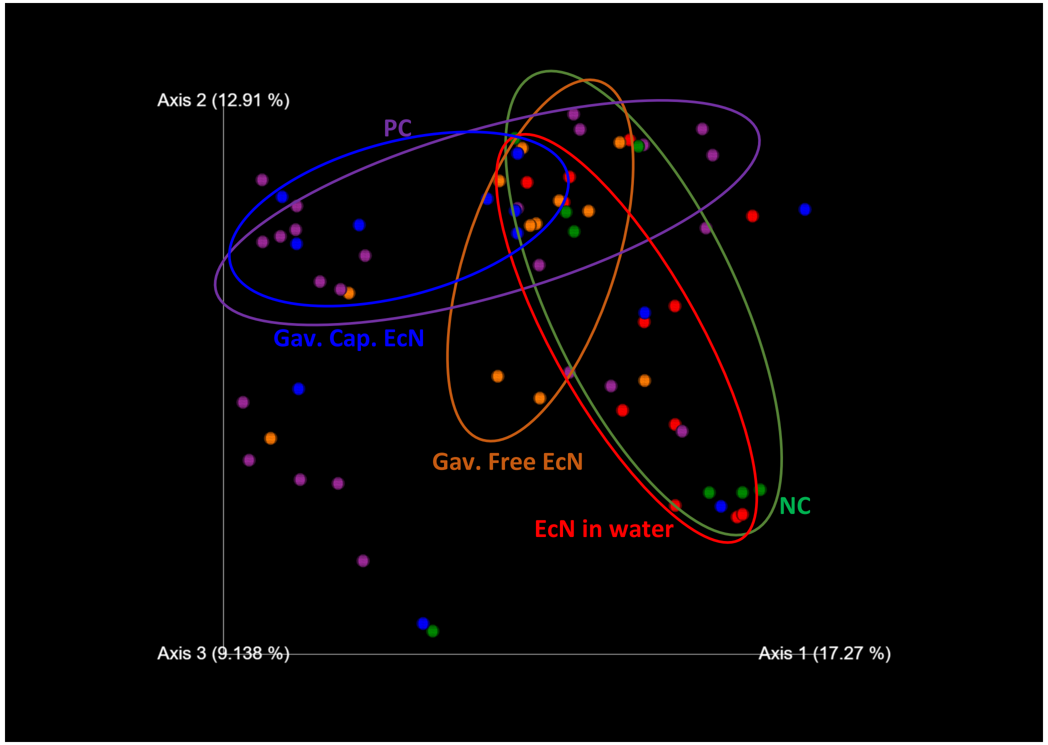
Axis 2 (12.91 %)
PC
Gav. Cap. EcN
Gav. Free EcN
EcN in water
NC
Axis 3 (9.138 %)
Axis 1 (17.27 %)

Figure S3
